# Identifying and predicting Parkinson’s disease subtypes through trajectory clustering via bipartite networks

**DOI:** 10.1371/journal.pone.0233296

**Published:** 2020-06-17

**Authors:** Sanjukta Krishnagopal, Rainer von Coelln, Lisa M. Shulman, Michelle Girvan

**Affiliations:** 1 University of Maryland College Park, College Park, MD, United States of America; 2 University of Maryland School of Medicine, Baltimore, MD, United States of America; 3 Santa Fe Institute, Santa Fe, New Mexico, United States of America; Nathan S Kline Institute, UNITED STATES

## Abstract

Chronic medical conditions show substantial heterogeneity in their clinical features and progression. We develop the novel data-driven, network-based Trajectory Profile Clustering (TPC) algorithm for 1) identification of disease subtypes and 2) early prediction of subtype/disease progression patterns. TPC is an easily generalizable method that identifies subtypes by clustering patients with similar disease trajectory profiles, based not only on Parkinson’s Disease (PD) variable severity, but also on their complex patterns of evolution. TPC is derived from bipartite networks that connect patients to disease variables. Applying our TPC algorithm to a PD clinical dataset, we identify 3 distinct subtypes/patient clusters, each with a characteristic progression profile. We show that TPC predicts the patient’s disease subtype 4 years in advance with 72% accuracy for a longitudinal test cohort. Furthermore, we demonstrate that other types of data such as genetic data can be integrated seamlessly in the TPC algorithm. In summary, using PD as an example, we present an effective method for subtype identification in multidimensional longitudinal datasets, and early prediction of subtypes in individual patients.

## Introduction

Parkinson’s disease (PD) is the second most common neurodegenerative disorder, affecting an estimated 7-10 million people worldwide [1]. The cause of PD is unknown, and the disease course is variable with age of onset and rate of progression differing across the population [2]. Furthermore, the clinical presentation is variable, with a broad range of possible motor and non-motor symptoms [3]. Based on these differences, multiple PD subtypes have been proposed, based on clinical intuition or unbiased data-driven approaches like cluster analysis [4]. Disease subtypes, which are likely to differ by the underlying etiology, treatment responsiveness and prognosis, will therefore facilitate PD research, management, and counseling of patients regarding prognosis [5, 6].

There is currently no consensus on Parkinson’s subtypes that are biologically valid and clinically relevant, and the best approach for identifying such subtypes remains elusive [7]. Lack of integration of longitudinal data for a large number of variables and lack of data-based prognoses are limitations of existing approaches [8].

Network medicine [9–13] offers a promising approach for untangling the complexities due to multiple influences on disease manifestation and progression via analysis of interconnections within data. For example, studies of the human disease network (i.e. the ‘diseaseome’) [13], in which diseases are linked if they share one or more associated genes, are useful for identifying disease pathways and predicting other disease-related genetic variants [11]. With few exceptions, most network medicine studies have focused on biomolecular data [13–16] rather than the complexities of clinical phenotypic assessments, and disease subtyping based on disease progression patterns is relatively unexplored [17, 18]. Another possible benefit of network medicine approaches is that they offer ways to integrate different types of data, for example to simultaneously incorporate clinical assessments with genetic data. This is especially important for PD, as a large number of genetic variants have been identified as risk factors [19]. Further, evidence has emerged that the same genetic risk variants also determine certain clinical features of the disease, highlighting the need to explore novel approaches that integrate genetic data into clustering (or subtyping) algorithms [20, 21].

Technological innovations in data processing and storage capacity have enabled development of large clinical datasets, containing longitudinal clinical and biological data. In this work we use data from the Michael J. Fox Foundation’s Parkinson’s Progression Markers Initiative (PPMI), a worldwide study to establish a comprehensive set of clinical, imaging and genetic data (http://www.ppmi-info.org). Such datasets require sophisticated data-driven approaches for effective extraction and analysis of clinically relevant information. Data-driven methods are typically applied to diseases in two ways: disease-specific, i.e., identifying disease subtypes and variable progression patterns from large scale patient data, and patient-specific, i.e., predicting disease subtype and trajectory in the individual patient based on their data. Our work incorporates both these perspectives and presents a network science method that not only identifies disease subtypes using diverse types of patient data (e.g., genetic and clinical variables), but is also predictive. We present our results based on a PD dataset, however this method is easily applied to other chronic medical conditions.

To provide an intuitive data-driven solution that is both disease- and patient-centric, we develop the novel Trajectory Profile Clustering (TPC) algorithm to identify PD subtypes through similarities in patterns of progression. Additionally, we demonstrate the predictive ability of our algorithm on a test/validation cohort of new patients. We also explore inclusion of four PD genetic variants in our approach, to demonstrate its capacity to simultaneously incorporate clinical, demographic, and genetic information. Thus, TPC is a data-driven algorithm that can incorporate different types of data (e.g., genetic, clinical etc.) and different weighting schemes for different variables in order to cluster patients according to the similarity of their disease progression. In addition, TPC also offers predictive power, making it a useful tool for clinicians in the study of multivariate, progressive disease datasets. Our method, to the best of our knowledge, presents a new and easily generalizable approach for robust subtype identification by accounting for disease progression patterns in addition to overall variable profiles. This work is aimed at bridging the gap between the computational methodologies developed by network and data scientists and the clinical experience of health professionals.

## Materials and methods

### Description of data

**Fig 1:**
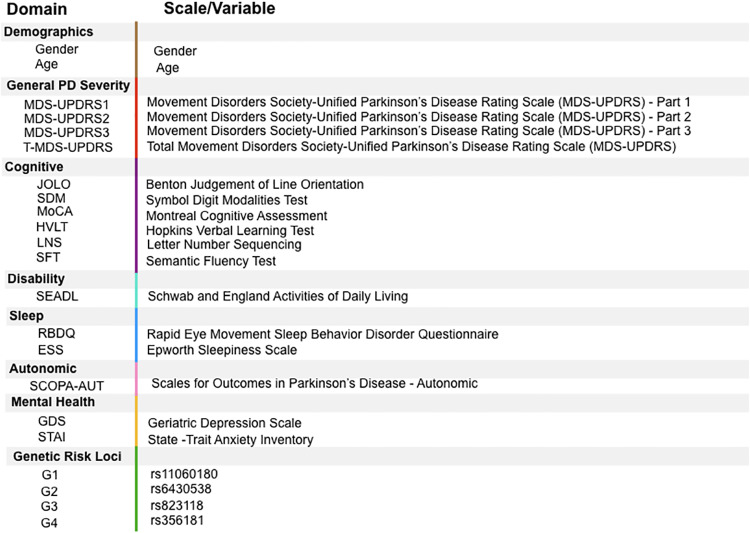
Description of PPMI Data. Data includes two demographic variables, outcome variables from six clinical domains, and four genetic single nucleotide polymorphisms.

Data used in the preparation of this article were obtained from the Parkinson’s Progression Markers Initiative (PPMI) database (www.ppmi-info.org/data). The data consists of patient variable values across 5 time points: baseline values (which we denote as year 0) and years 1,2,3, and 4. Of the 430 patients at baseline in this dataset, 314 patients remained in year 4. Once patients with incomplete data were excluded, 194 patients remained in our analysis. Twenty percent of this population (number of individuals *n* = 39) was kept as a test/validation dataset. The remainder of the patients (*n* = 155) formed the training dataset that was used in the algorithm to identify PD subtypes. The data included demographics (gender and age in year 4), clinical variables from six clinical domains (General PD Severity, Disability, Cognition, Autonomic Function, Sleep, and Mental Health) and 4 PD genetic variants (Fig 1). PPMI motor assessment was performed in a ‘practically defined off’ state, i.e., subjects are asked to withhold their medication prior to the assessment for 12 hours for a defined “OFF” medication score, practically eliminating medication effects on motor symptoms in this dataset.

### Trajectory Profile Clustering algorithm

**Fig 2:**
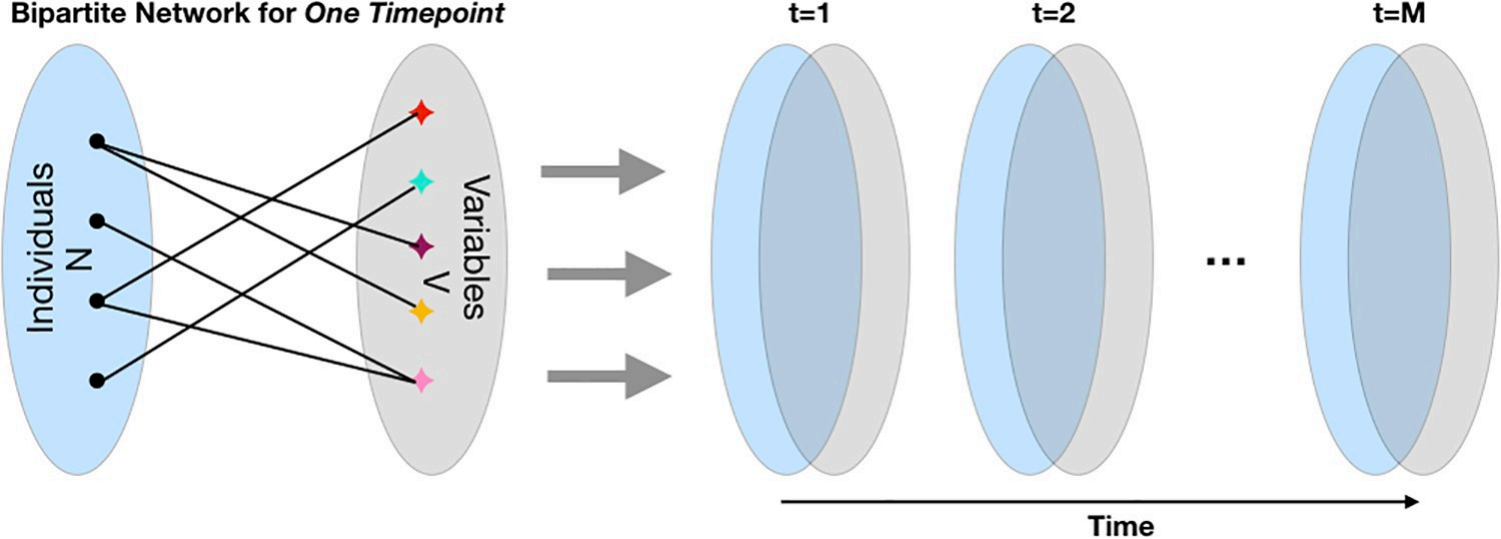
Stacking bipartite networks across time. An illustration of an individual-variable bipartite graph at one timestep (left). Set of bipartite graphs across time (right).

Our Trajectory Profile Clustering algorithm is designed to group together patients based on the similarities of their disease trajectories. The algorithm proceeds as follows:

1. *Create bipartite networks connecting individuals to variables*: At time point *t* (e.g., baseline, year 1, year 2, etc.) we construct an *N* × *V* bipartite graph modeling connections between individuals and disease variables, where *N* is the number of individuals in the training population and *V* is the total number of variables, as illustrated in Fig 2. For *M* time points, we can represent the set of these bipartite graphs as an *N* × *V* × *M* multidimensional array, where *X*_*ivt*_ gives the value of individual *i*’s disease variable *v* at time *t*.
2. *Transform data for variable uniformity*: For each non-binary variable, we determine its ‘direction’. For variable *v*, if higher values of the variable are associated with greater disease severity then its direction *d*_*v*_ = +1; otherwise *d*_*v*_ = −1. For our data, clinical variables ESS, RBDQ, GDS, STAI, UPDRS and age have *d*_*v*_ = 1, and HVLT, JOLO, SFT, LNS, SDM, MoCA, SEADL have *d*_*v*_ = −1. We then define a new *N* × *V* × *M* multi-dimensional array *Y* such that *Y*_*ivt*_ = *d*_*v*_ *X*_*ivt*_ for non-binary variables. For binary variables, *Y*_*ivt*_ = *X*_*ivt*_.
3. *Construct patient trajectory profiles*: For each patient *i*, we construct a *V* × *M* trajectory profile matrix, *T*^*i*^. The matrix entries of *T*^*i*^ are calculated as follows:
  - For non-binary variables:
    $$\begin{array}{cc} T_{vt}^{i} & =1\;\text{if}\;Y_{ivt}>\theta_{v}\\ & =0\;\text{otherwise.} \end{array}$$ (1)
    where *θ*_*v*_ is the threshold for variable *v*. In this manuscript, we set *θ*_*v*_ to the median baseline value of variable v in the training data. We threshold the connections, i.e., the individual is only connected with disease variables for which they have a high enough severity. This thresholding causes patients to be shown as unconnected to all variables in Fig 2.
  - For binary variables:
    *For gender*: $T_{vt}^{i}=1$ if the patient is male, $T_{vt}^{i}=0$ otherwise. *For genetic risk loci*: $T_{vt}^{i}=1$ if patient contains single nucleotide polymorphisms (SNP) *v*, $T_{vt}^{i}=0$ otherwise. Each SNP is treated as independent.
4. *Create a patient-patient network with connections based on trajectory similarity*: After having defined the trajectory profile matrix *T*^*i*^ for each individual *i*, we create a patient-patient network *P* of all patients in the training set. The nodes of this network correspond to patients and the strength of a link between patient *i* and patient *j* captures the similarity of their trajectory profiles. *P* has an adjacency matrix given by:
  $$\begin{array}{c} P_{ij}=\sum_{v,t}\left(T_{vt}^{i}\equiv T_{vt}^{j}\right). \end{array}$$ (2)
  In other words, *P*_*ij*_ gives the number of matrix entries for which trajectory profile *T*^*i*^ has the same value as *T*^*j*^. This formulation implies that variables are equally weighted. Other applications may require unequal weighting for variables and time points, in which case one may calculate the patient-patient matrix as follows: $P_{ij}=\sum_{v,t}w_{vt}\left(T_{vt}^{i}\equiv T_{vt}^{j}\right)$ where *w*_*vt*_ is the weight of variable *v* at time *t*. An alternate more finely resolved approach to constructing the patient-patient network *P* would be, for example, to divide the baseline data for each variable into quartiles. In this case, the strength of a link between two patients would be determined by the number of variable-timesteps for which their values landed in the same quartile. In preliminary investigations, the more finely resolved approach gave similar results, so for simplicity we focus in this manuscript on the median-based discretization scheme defined by Eq 1.
5. *Cluster the network to identify communities/subtypes*: We then perform Louvain community detection [22] to maximize the Newman-Girvan modularity function [23] on the uni-partite network defined by the weighted matrix P. As is common in network community detection approaches [24], the number of communities is not set a priori, but rather chosen so that the modularity is maximized. This process allows us to cluster trajectory profiles, and hence patients, into communities (subtypes) which are relatively densely connected.
6. *Construct aggregate profiles to characterize each community/subtype*: We average the trajectory profiles of all patients in each community *C*^*l*^ to obtain the ‘community/subtype profile’ *S*^*l*^. The subtype profile is indicative of the variable features that describe the subtype. More specifically, it is the normalized average of the trajectory profiles of all the patients in that subtype, i.e., *S*^*l*^ is a *V* × *M* matrix with elements defined by
  $$\begin{array}{c} S_{vt}^{l}=\frac{\sum_{i\in C^{l}}T_{vt}^{i}}{N_{l}U_{v0}} \end{array}$$ (3)
  where *N*_*l*_ is the total number of individuals in community *C*^*l*^. *U*_*v*0_ is a normalization constant that represents the average value for variable v in the baseline: $U_{v0}=\frac{\sum_{i}T_{v0}^{i}}{N}$, and 0 denotes the baseline year.

### Prediction scheme for test patients

From baseline data, we predict the community/subtype that an individual test patient (patient whose data was not used in identifying the PD subtypes) belongs to. We then check whether the test patient is still aligned with the same community/subtype after 4 years to demonstrate the utility of our baseline prediction.

To predict test patient *i*’s subtype from his/her baseline profile, we find the community (subtype) *C*^*l*^ whose baseline community profile, with elements $S_{v0}^{l}$, has the smallest Euclidean distance from the patient’s baseline profile. In other words, *l* is chosen to minimize the distance

$$\begin{array}{c} d_{0}^{il}=\sqrt{\sum_{v}{\left(T_{v0}^{i}-S_{v0}^{l}\right)}^{2}}. \end{array}$$ (4)

Does the patient’s trajectory match the subtype’s trajectory? We then investigate the quality of the subtype/community baseline prediction at a later time *t* by calculating the patient’s subtype/community *C*^*l*′^ is chosen to minimize the distance between the community profile and the patient’s profile at time *t*:

$$\begin{array}{c} d_{t}^{il^{\prime }}=\sqrt{\sum_{v}{\left(T_{vt}^{i}-S_{vt}^{l^{\prime }}\right)}^{2}}. \end{array}$$ (5)

The prediction accuracy is then defined as the fraction of test patients for which the subtype identification (*l*) from the baseline matches the subtype identification (*l*′) at a later time *t*.

## Results

### TPC algorithm for PD subtype identification

In this section, we present the disease subtypes (communities) identified by our method from the training patient data. Maximizing Newman-Girvan modularity on the patient-patient trajectory profile network gives us three distinct subtypes, i.e., three is the optimal number of subtypes for this data, as indicated by the modularity measure.

**Fig 3:**
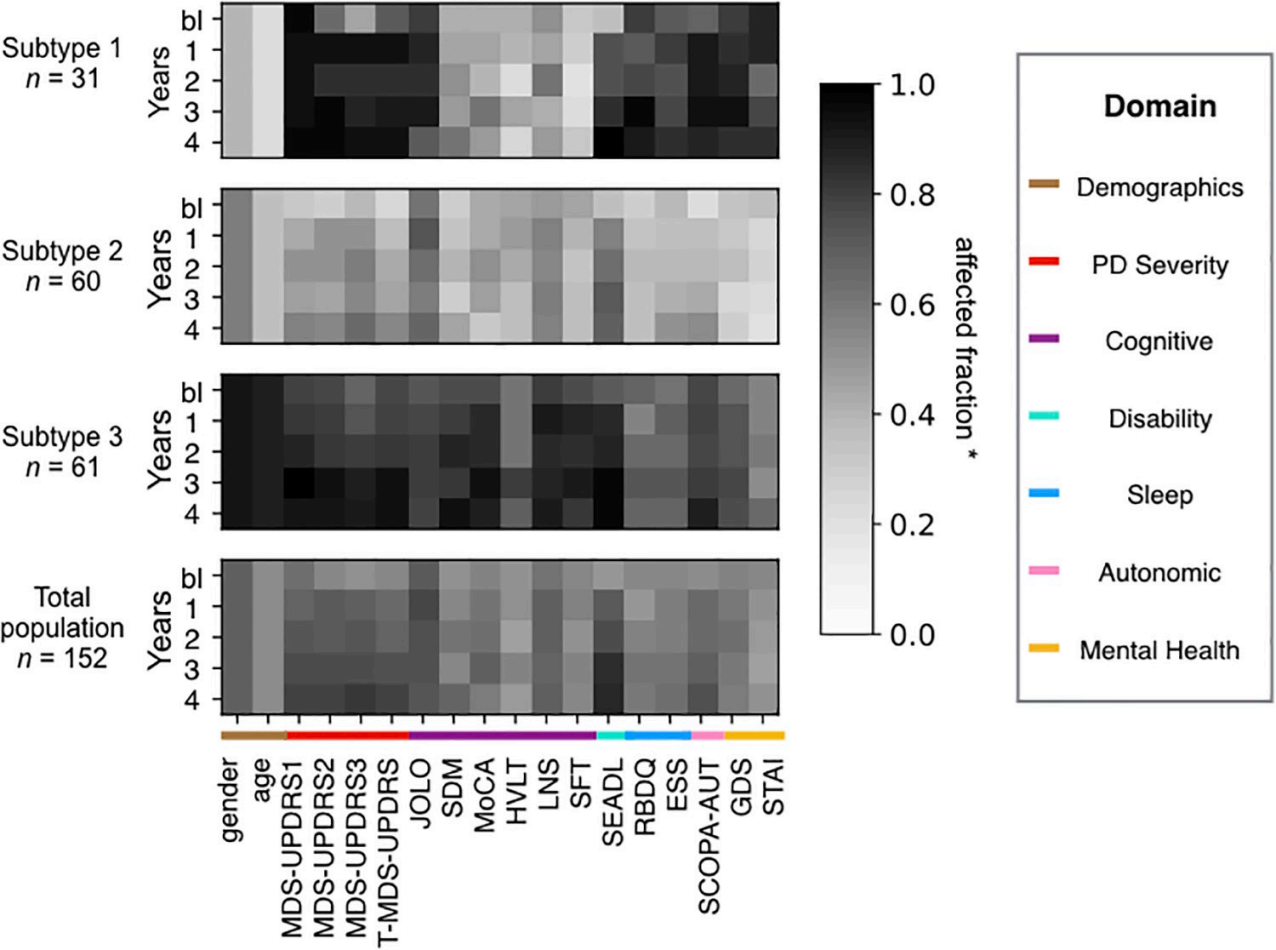
Variable profiles of the Parkinson’s subtypes identified by the TPC algorithm. Subtypes/communities identified by our algorithm: top three panels show three subtype/community profiles (average profile of all patients in the subtype). Subtypes identified by the algorithm containing fewer than 10 patients are not shown (3 patients fall under this category). The bottom panel shows the total population profile. The shade of grey indicates the affected fraction, i.e, fraction above baseline median in the direction of disease progression for the continuous variables, and fraction that is male for gender. n is the number of patients in the subtype. The variable names are listed below the panels (See Fig 1 for description).

The clinical profiles plus demographics of each subtype as compared to the entire study population are shown for baseline and years 1-4 in Fig 3. The darkness of the shade of grey of a continuous variable in a year denotes the fraction of the subtype population that has a value above the median of the total population baseline for that variable. The darkness of the shade of grey for a binary variable is the fraction of the subtype population containing that variable (male in the case of the variable gender). In the raw data, a higher raw score in some variables (such as the Montreal Cognitive Assessment) implies a healthier/less severely affected patient, while for other scales, the opposite is true (higher score = greater severity). Therefore, in step one of our algorithm we transformed the data, so that for all variables except for the genetic and demographic variables, a higher score is associated with greater severity of that variable and a deeper shade of grey.

### Description of the subtypes

As seen in Fig 3, the subtypes can be outlined as follows:

- Subtype 1 is the ‘mixed subtype’, with a striking discrepancy between severe impairment of motor and autonomic function, mental health and sleep on the one hand, and good cognition on the other hand, both at baseline and over time, and also young and predominantly female;
- Subtype 2 is the ‘mild subtype’, with milder than average impairment in all domains (motor, cognitive, autonomic and mental) at baseline and throughout the study duration (age and gender distribution close to the average of the entire population);
- Subtype 3 is the ‘severe subtype’, with worse than average impairment in all domains, in particular motor and cognition. This subtype is also predominantly male and older than average. Autonomic and mental function is less impaired than in the mixed subtype (subtype 1)

The bottom panel in Fig 3 shows the profile of the total population. Since the threshold variable severity in an individual is set with respect to the median of the total population at baseline, the total population baseline profile for all variables has a value close to 0.5 (i.e., 50% of the total population at baseline has a value of 1 for any variable, and the other half has a value of 0). Fluctuations of the baseline total population value around 0.5 occur when multiple people in the population have a value coinciding with the baseline median. In the Appendix A, we provide statistical analyses comparing the subtypes at the baseline and the final timepoint (year 4). These analyses support the subtype descriptions provided above.

### Early prediction of patient subtypes

**Fig 4:**
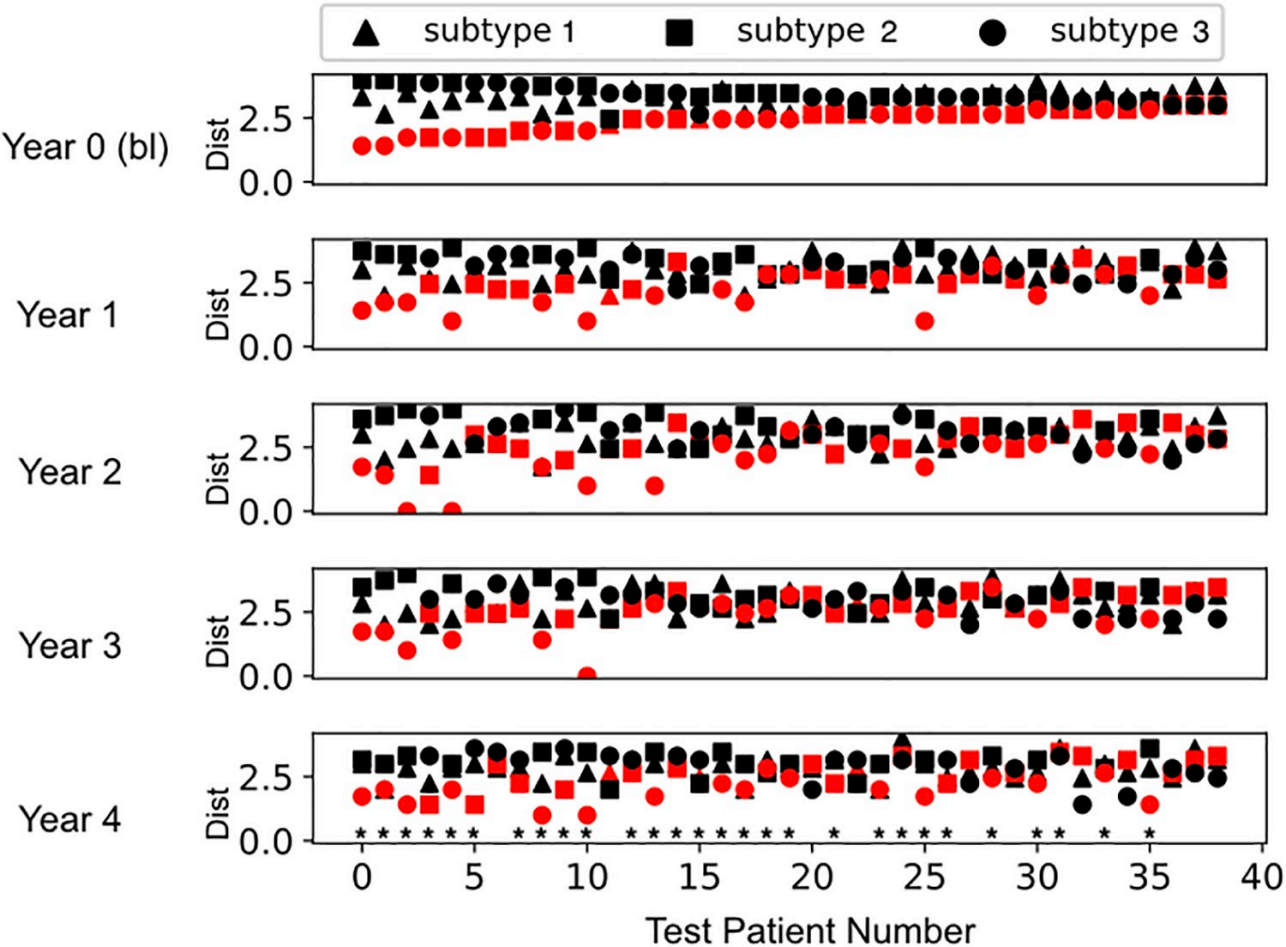
Prediction of test patients into the subtypes. The *i*^*th*^ panel (row) shows the distance between the test patient *i*^*th*^ year profile and the *i*^*th*^ year subtype profile (shape coded). The predicted subtype for each individual (subtype with minimum baseline-year distance) is colored red to allow for tracking across the years (panels). Prediction accuracy in year 4 is 72%. Patients whose year 4 subtype is correctly predicted from their baseline data are designed by a star. Data includes 39 test patients and 18 clinical variables across 5 time points: baseline (bl) or year 0 + years 1,2,3,4).

In addition to identifying PD subtypes, our method predicts the individual patient subtype years in advance. In this section we use the test patient cohort (*n* = 39) to assess the accuracy of early prediction of disease subtype. Data from these test patients was not used in the identification of the subtypes. Fig 4 shows the prediction of future PD subtype based on baseline data for 39 test patients that run across the horizontal axis. The top panel shows the Euclidian distance between the baseline profile of a patient and the baseline profile of each subtype (subtypes are shape coded). The subtype with which the patient has minimum *baseline* distance is the ‘predicted subtype’, and is marked in red. Patients are organized from left to right in order of decreasing confidence, i.e., from minimum to maximum distance of the patients’ baseline profile with the predicted subtype baseline profile. The remaining panels follow the same plotting scheme for consecutive years. The red-coding of baseline predicted community makes it easy to track across the years. Finally, in year 4, we assess the accuracy of our predictions by identifying the ‘actual final subtype’ (the subtype with minimum distance to the patient in year 4). If the actual final subtype and the predicted subtype are the same, then we consider our prediction to be successful for that patient. In other words, for a patient, if in year 4 the red subtype has the minimum distance (is below the black subtypes) then our prediction is successful. For the test PD patients in the PPMI dataset, our algorithm uses only their baseline year data to predicts their PD subtype after 4 years of disease progression with 72% accuracy.

### Incorporating genetic data into the TPC algorithm

Genetic variants are increasingly recognized as important determinants of disease subtype and disease progression and prognosis. As an exploratory objective, we investigated the integration of genetic variants (single nucleotide polymorphisms, or SNPs) in previously identified PD risk loci into our TPC-based approach. Each patient has 2 copies for each piece of genetic information, and there are by definition 2 variants for each SNP. Hence, there are 3 possible combinations of the 2 variants for each of the genetic risk loci. PPMI contains information for 28 such SNPs for each patient. As a proof of principle, we selected 4 of those 28 SNPs to be included in our approach. For one of those SNPs (rs356181/2, labeled ‘G4’ in our study), an association with PD motor has recently been described [25], making this an obvious choice for our study. Recently, genotype-phenotype correlations have been described for a number of SNPs associated with PD risk [21, 26, 27]. However, there was minimal overlap in terms of which genetic variants were associated with specific clinical features of PD, even though two of these studies were performed by the same consortium, analyzing data from essentially the same collection of large PD cohorts [21, 27]. Consistent with our proof-of-principle approach, we therefore picked the additional 3 SNPs based on their high minor allele frequency, so that all 3 possible combinations of the 2 genetic variants were present in sufficient numbers in our study population of 194 subjects. The G allele of one of these SNPs (rs1106180, labeled ‘G1’ in our study) is associated with a later age of onset [21].

**Fig 5:**
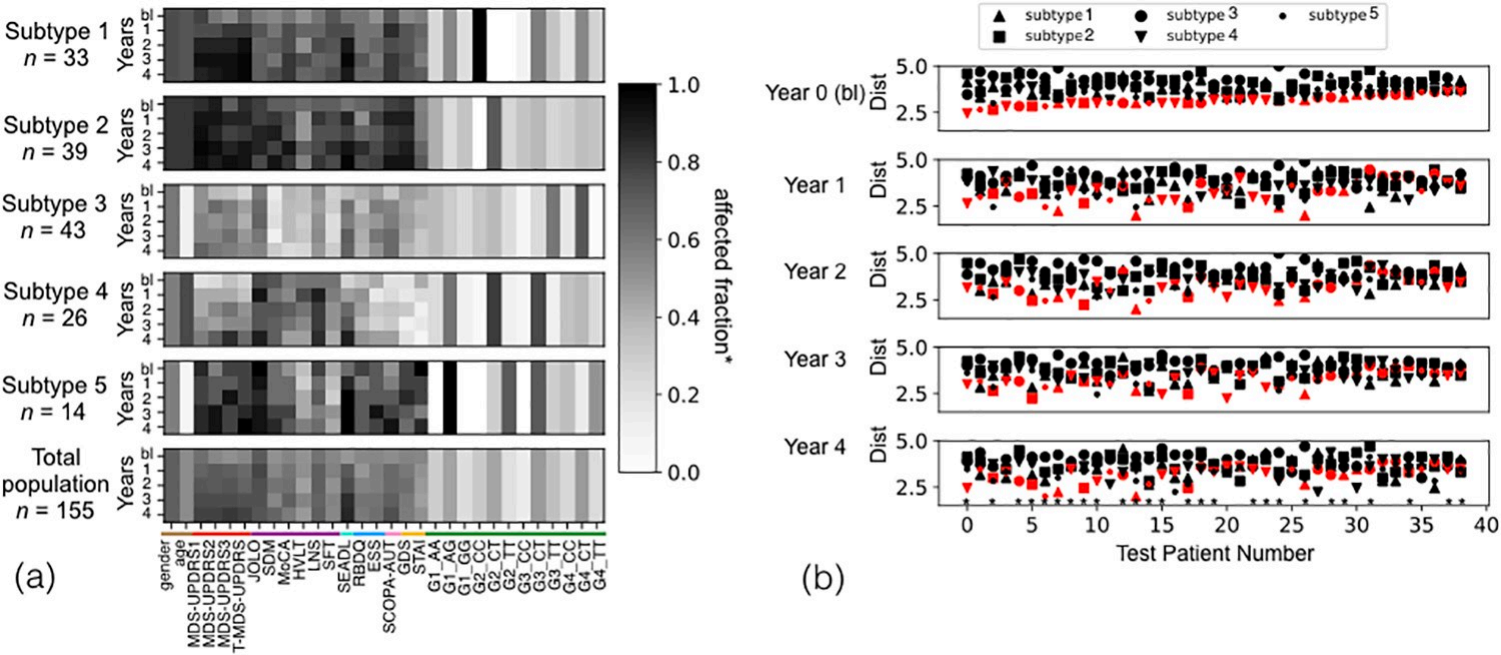
Variable profiles and test patient subtype prediction using clinical and genetic data. (a) Top five panels show five average community (subtype) profiles, identified by our TPC algorithm. The bottom panel shows the total population profile. The legend is a measure of the affected fraction, i.e, fraction above baseline median in the direction of disease progression for the continuous variables, and fraction that is male and fraction containing the genetic SNP for gender and genetic variables respectively. n is the number of patients in the community. (b) The *m*^*th*^ panel shows the distance between the test patient *m*^*th*^ year profile and the *m*^*th*^ year profile of the subtypes (shape coded). The predicted subtype for each individual (subtype with minimum baseline distance) is colored red to allow for tracking across the years (panels). Prediction accuracy in year 4 is 67%. Patients whose year 4 subtype is correctly predicted from their baseline data are designed by a star. Data includes 39 test patients and 18 clinical variables across 5 time points: baseline (bl) or year 0 + years 1,2,3,4.

Fig 5 shows the five subtypes identified when genetic data is introduced. Here, the number of subtypes is determined by maximizing the network modularity measure for the network created using clinical and genetic information. The plots Fig 5(a) and 5(b) are organized in the same way as Figs 3 and 4 respectively. In Fig 5(a), the darkness of the shade of grey of a variable in a year denotes the fraction of the subtype that has a value above the baseline median of the total population of that variable, and the color coding on the x axis denotes the domains as in Fig 3.

Subtypes 1 (top) and 2 have relatively similar clinical profiles, with the difference being in their genetic profile. The CC genotype of G2, and CT genotype of G3 and G4 are frequent in subtype 1, and the CT is the most common genotype of G2 in subtype 2. Subtypes 3 and 4 relatively mild symptoms at baseline, and an overall benign progression of most variables over the course of 4 years. Subtype 3 includes more patients who are younger and less cognitively impaired than average. Subtype 4 has less psychiatric, autonomic and sleep impairments but intermediate motor impairments (MDS-UPDRS3). Finally, subtype 5 is small (*n* = 14) and young with rather severe symptoms at baseline and rapid progression across most clinical domains. Each of the subtypes has a distinct genetic profile. When genetic data is added to the analysis, baseline prediction of patient subtype in the test group 4 years later shows an accuracy of 67%.

## Discussion

Multidimensional clinical datasets are valuable resources that are not used to their full potential due to the analytic challenges of diverse biomarkers and outcome variables. We describe development of a method to identify disease subtypes based on the pattern of progression of multidimensional clinical data including demographics, clinical variables, and genetics. We then validate our method by measuring the accuracy of subtype prediction in individual patients based on baseline clinical and genetic variables. The disease subtypes are characterized by patterns of progression of the clinical variables. The concordance between our results with the domain-structure of the variables supports our approach. For example, in the clinical-only case, subtypes 1 and 3 have high progression of all PD severity variables and subtype 2 has a low progression of all PD severity variables. Variables within other domains such as Sleep, Mental Health and Cognition also show common intra-domain patterns within a subtype.

Our predictions of the future subtype of individual patients in the test sample based on their baseline data, shows good accuracy in predicting disease subtypes four years later (72% for clinical data and 67% for clinical+genetic data). 4 years is a significant time-scale for PD, which has large subtype variability. Our prediction accuracies 4 years in advance are very promising in the field of PD medicine. The explanation for the reduction in predictive accuracy with addition of genetic data may be due to: 1) the inclusion of a very limited number of genetic risk loci, 2) that genetic data isn’t predictive of PD subtype within the 4-year time frame of our data or 3) that the genetic data has a large variance in the population, thus requiring a larger dataset for long-term prediction (the larger number of subtypes found by our method may indicate this). Nonetheless, from a methodological perspective, this exploratory work successfully demonstrates the inclusion of genetic data. Other biomarkers (i.e. serologic and cerebrospinal fluid biomarkers) can also be easily integrated into our analysis. Our algorithm is likely to benefit from more extensive datasets with larger populations.

A number of studies have identified PD subtypes based on baseline characteristics [7, 28–30]. In contrast to that, our innovative algorithm uses longitudinal data (or the trajectory of the different variables over time) to identify disease subtypes. In other words, our method accounts for both disease variable values as well as their progression patterns. To our knowledge, this is a novel approach. The baseline features of individual patients in a test cohort were then used to predict their future disease trajectory (prognosis). Our study represents an innovative network-based data-driven approach, that has advantages over previous methods by taking full advantage of large heterogenous, longitudinal datasets.

Despite the fact that genetic factors likely play a major role in determining PD subtypes [25], few data-driven algorithms for suptype identification exist that incorporate genetic data. Two recent studies have developed models of PD progression based on clinical, demographic and genetic data at baseline, using hierarchical cluster analysis and a Bayesian multivariate predictive inference platform, respectively, to identify PD subtypes that show significant differences in their rate of progression over time [7, 26]. Even though both of these studies thoroughly evaluate the differences of baseline subtypes in terms of long-term outcome, neither of them determines the prediction accuracy of their baseline subtype classification by repeating the subtype classification algorithm at the last time point of the follow-up period. The authors of one of the two studies [26] used the coefficient of determination R2 as a measure of overall explanatory power of their model and found it to be 41% in the study cohort, and 9% in an independent validation cohort. However, this is a measure of how well the baseline data explain the variability at follow-up when applying their model, rather than a metric of the accuracy of subtype prediction that we introduced in our study as a novel and, in our opinion, critically important quality metric that may serve as reference when comparing our results with future subtype classification algorithms.

Our trajectory clustering method works with various types of data including clinician- and patient-reported outcome measures, genetics, physical performance measures, as well as diverse results from diagnostic investigations. This analysis uses demographics, clinician- and patient-reported data, and genetic data. In our analysis, each genetic SNP (if considered) and clinical variable is treated independently and allotted the same weight. Our algorithm allows for variable weightings, where each domain and SNP is assigned a chosen weight. However, this raises the question of how the weighting would be decided. For example, if we had allotted equal weights to one hundred SNPs in our analysis in addition to the 18 clinical variables, the genetic information would dominate the algorithm, and affect the resulting communities. On the other hand, different weighting strategies may be preferable based on the study aims. For example, if the main objective is to identify disease subtypes based on motor vs. cognitive function, one could allot equal cumulative weight to the motor and cognitive domains.

A strength of our algorithm, which is also a caveat, is that it is entirely data-driven. The level of severity of each variable relative to the baseline median is used to normalize all variables, as opposed to the absolute value of the variable. This is done to readily compare changes in different variables. A notable example is the clinical variable, SEADL (a disability scale). SEADL is a relatively insensitive scale in the early years of PD since there is little functional disability in the years following diagnosis. Yet, in our analysis SEADL shows high progression (darker shade in later years) in Figs 3 and 5(a). It is important to note that this dark shade isn’t indicative of the absolute severity. It only tells us that a larger fraction of the population in the later years has SEADL values above the baseline median of the total training population (which may be low to begin with). Like the results from any data-driven approach to identify disease subtypes, our results should be applied in practice in conjunction with medical expertise. An additional limitation of our approach is the fact that a number of choices had to be made by our team of data scientists and clinicians to create this algorithm including thresholds, the weighting scheme for all variables in the network, and variables to include. While our data-driven method is primarily agnostic, these choices are inevitably somewhat arbitrary in nature, and will have an impact on the result of the analysis. Furthermore, they may be different for different applications/datasets. Lastly, like any data-driven method, the robustness of the method is proportional to the quantity of data. Hence, while this method is suitable for heterogenous datasets such as the PPMI data, there may be other datasets that have large gaps in data collection, inconsistent times of acquiring data, too much variation in data or simply too little data- therefore, our method may not be suitable for all clinical datasets.

Our approach is innovative, adaptable, and clinically relevant. PD subtyping [31] is an area of active research but there are currently no clinically prognostic analyses in use for the management of PD. Application of an approach like ours for subtype identification as a predictive model of PD progression will help the neurologist improve clinical management of individual patients. For example, such an approach may prompt the clinician to pursue earlier, more aggressive management for those patients for whom the algorithm predicts a more rapid disease progression (i.e., ‘precision medicine’). It may also guide the neurologist to perform targeted investigations (e.g. cognitive testing) in individuals based on their subtype. Finally, prediction of disease progression will improve prognostic counseling, a problem commonly encountered by clinicians, by bringing to attention disease features that are predicted to develop over the course of the disease. A natural extension of this work will be to implement this method for datasets in other chronic medical conditions. Other promising future directions include extending the TPC algorithm to incorporate and compare other network clustering approaches, such as multi-layer network clustering [8]; studying the effect of treatment on progression of disease variables, and predicting modifications of algorithm-identified subtypes as a consequence of different treatments.

## A statistical analyses

We conducted statistical tests to validate our approach and demonstrate some of the differences between the subtypes identified by our TPC algorithm. These tests were focused on differentiating between the 3 subtypes illustrated in Fig 3: mixed, mild, and severe.

**Fig 6:**
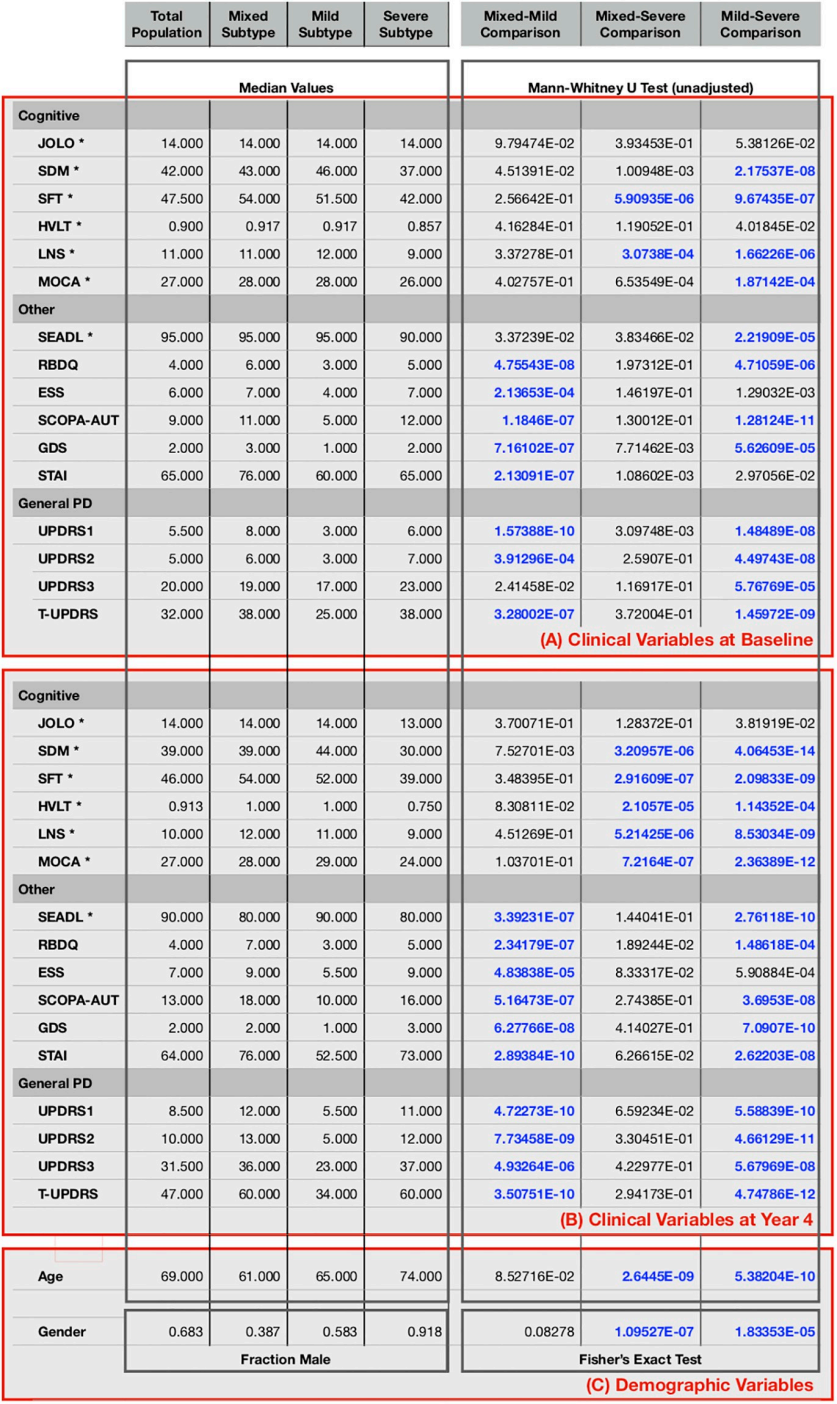
Statistical analysis. Statistical analysis comparing the 3 subtypes described in the main text: mixed, mild, and severe. Features of the total population are also listed. Medians are calculated from the raw data. Variables with negative directions are denoted by an asterisk (*). Comparisons meeting our criteria for statistical significance are shown in bold blue text. The top box (A) provides statistics for the baseline clinical variables, the middle box (B) for the year 4 clinical variables, and the bottom box (C) for demographics.

For each pair of subtypes, for each non-binary variable, we conducted a Mann-Whitney U test (implemented in SciPy [32]), which allows 2 groups to be compared without assuming that values are normally distributed. This test was chosen because values for many of the variables violated the normality assumption. For gender, which was binary in our analysis, we performed Fisher’s exact test to calculate two-sided p-values. Fig 6 reports unadjusted p-values from these tests applied at two different time points: the baseline and the final timepoint (year 4). To achieve an overall significance level of *α* = 0.05, we used the conservative Bonferroni correction to account for Type I errors due to multiple comparisons, giving us an adjusted significance level of *α*_adjusted_ = 0.05/*n*_*c*_ ≈ 5*e* − 4, where *n*_*c*_ = *n*_*p*_(*V*_*d*_ + 2*V*_*e*_) = 102 is the total number of comparisons, *n*_*p*_ = 3 is the number of pairs of subtypes, *V*_*d*_ = 2 is the number of demographic variables (age and gender), and *V*_*e*_ = 16 is the number of potentially evolving clinical variables that we assess at each of the 2 timepoints. Comparisons meeting our adjusted significance criteria are highlighted in bold in the figure.

For the baseline clinical values, we see several significant statistical differences between subtypes that support the descriptions presented in the main text. For example, compared with both the mixed and severe subtypes, the mild subtype shows significantly lower general PD severity according to 3 of 4 of the PD scales (UPDRS1, UPDRS2, and TUPDRS) as well as lower impairment according to several other scales, including the GDS (depression), SCOPA (autonomic) and RBDQ (sleep) scales. Compared with patients in the mild subtype, patients in the severe subtype show significantly greater impairment in 4 of 6 cognitive scales: SDM, SFT, LNS, and MOCA, as well as in the SEADL (disability) scale. In addition, compared even to patients in the mixed subtype, patients in the severe subtype show significantly greater impairment in the SFT and LNS cognitive scales.

For year 4 clinical values, we see even more significant differences between the subtypes than at baseline. For example, compared with the mixed and severe subtypes, the mild subtype shows significantly less impairment for all 4 PD severity scales, up from 3 at baseline. Further, the severe subtype is significantly more impaired than *both* the mixed and mild subtypes according to 5 of the cognitive scales, compared to just 2 at baseline.

We also see a significant difference in demographic variables between the subtypes. Compared to the other 2 subtypes, the severe subtype has older patients and a greater fraction of males.

While these statistical tests only compare the subtypes at two timepoints, they serve to validate our approach by highlighting some of the significant differences.
